# Combined Effect of Ultrasound and Low-Heat Treatments on *E. coli* in Liquid Egg Products and Analysis of the Inducted Structural Alterations by NIR Spectroscopy

**DOI:** 10.3390/s22249941

**Published:** 2022-12-16

**Authors:** Dávid Nagy, László Baranyai, Lien Le Phuong Nguyen, Andrea Taczman Brückner, Tamás Zsom, Csaba Németh, József Felföldi, Viktória Zsom-Muha

**Affiliations:** 1 Department of Food Measurement and Process Control, Institute of Food Science and Technology, Hungarian University of Agriculture and Life Sciences, Somlói út 14–16, H-1118 Budapest, Hungary; 2 Department of Livestock Product and Food Preservation Technology, Institute of Food Science and Technology, Hungarian University of Agriculture and Life Sciences, Ménesi út 43–45, H-1118 Budapest, Hungary; 3 Institute of Biotechnology and Food Technology, Industrial University of Ho Chi Minh City, Ho Chi Minh City 700000, Vietnam; 4 Department of Food Microbiology, Hygiene and Safety, Institute of Food Science and Technology, Hungarian University of Agriculture and Life Sciences, Somlói út 14–16, H-1118 Budapest, Hungary; 5 Department of Postharvest, Supply Chain, Commerce and Sensory Science, Institute of Food Science and Technology, Hungarian University of Agriculture and Life Sciences, Ménesi út 43–45, H-1118 Budapest, Hungary; 6 Capriovus Kft., H-2317 Szigetcsép, Hungary

**Keywords:** egg, yolk, albumen, ultrasonic, preservation, quality

## Abstract

In this study, sonication with mild heat treatment was used to reduce the *E. coli* count in inoculated liquid whole egg, egg yolk and albumen. Ultrasonic equipment (20/40 kHz, 180/300 W) has been used for 30/60 min with a 55 °C water bath. The combination of sonication and low-heat treatment was able to reduce the concentration of *E. coli* from 5-log CFU × mL^−1^ below 10 CFU × mL^−1^ at 300 W, 40 kHz and 60 min of sonication in liquid egg products. The 60 min treatment was able to reduce the *E. coli* concentration below 10 CFU × mL^−1^ in the case of egg yolk regardless of the applied frequency, absorbed power or applied energy dose. The 30 min treatment of sonication and heating was able to reduce significantly the number of *E. coli* in the egg products, as well. Our results showed that sonication with mild heat treatment can be a useful technique to decrease the number of microorganisms in liquid egg products to a very low level. Near-infrared spectroscopy was used to investigate structural changes in the samples, induced by the combined treatment. Principal component analysis showed that this method can alter the C-H, C-N, -OH and -NH bonds in these egg products.

## 1. Introduction

Liquid egg products are highly nutritious, widely used (especially in the food industry) and more and more frequently consumed, but they are rapidly perishing food products. To ensure a safe product with a prolonged shelf life, egg liquids are needed to be treated against undesired microbial growth in some way. It is important to choose a treatment that does not damage the valuable substances in the egg and effectively reduce the number of microbes. Gentle heat treatment combined with sonication offers a suitable method for this important purpose, as it is able to induce the destruction of microbes at low temperatures (below 55 °C). In addition to the microbial count reduction in the different liquid egg forms, it is important to investigate the changes in the structure of the egg liquid that occur during and as a consequence of this non-thermal treatment.

Ultrasound is an environment-friendly green technology that can complete or substitute several heat-based conventional technologies [1]. Ultrasonication results in better quality, such as improved purity and enhanced organoleptic properties. It was found to contribute effectively to meat tenderization and curing, microbial inactivation, sterilization and pasteurization [1]. On the other hand, high-power ultrasound may result in off-flavor and odor appearance, a reduction in total phenolic content and protein denaturation due to cavitation [1]. Consequently, the application of low-power treatment is desired to avoid negative side effects of off-flavor and odor. Additionally, ultrasonication has been recently applied in combination with other techniques. Although specific *E. coli* strains can be used to an advantage in some industries [2], it is considered to be strictly hazardous from a food safety point of view. *Escherichia coli* was inoculated to tryptic soy broth and the lethal effect of ultrasound treatment was evaluated in a combination of 40–61 °C and 100–500 kPa [3]. The combination of lethal factors was found to significantly shorten the treatment time required to achieve a 5-log reduction in the quantity of survival microorganisms [3]. Ultrasound treatment of 2.39–20.96 W cm^−2^ with 30–120 min treatment time was tested on *E. coli* and *Bacillus cereus* [4]. The study revealed that treatment can result in the disruption of cell membrane integrity and inactivate targeted microorganisms during the beef curing process. A similar improved effect was observed for *E. coli*, *Bacillus cereus* and *Penicillium expansum* with additional chemicals on dried figs [5]. The combination of ultrasound and mild heat treatment was found to be successful in the inactivation of *Listeria monocytogenes* in raw milk and *E. coli* in apple cider up to a 5-log reduction [6]. A combination of heat and low-pressure treatments as 400 kPa with 55 °C, 100 kPa with 59 °C and 400 kPa with 59 °C were all found to be successful in a 5-log reduction of *E. coli* in apple cider [7]. Ultrasonication in the range of 20–60 W was found to improve the effect of antimicrobial peptide against *Listeria monocytogenes* [8]. A similar synergistic effect was observed between low-frequency ultrasound and antioxidants [9]. Liquid egg products, widely used by the food industry, are ideal materials for ultrasonic treatment purposes. They may have a high risk of contamination, while denaturation of albumen is an undesired quality alteration. High-power treatment of 968 W cm^−2^ at 35 °C for 20 min was proven to be successful against *Salmonella typhimurium* in liquid whole egg [10]. Ultrasound treatment at 20 kHz and the energy range of 24.6–42 W in combination with high hydrostatic pressure (HHP) treatments of 250–300 MPa resulted in a synergistic effect on nisin [11]. The combined treatment was able to achieve a 5-log reduction of *Listeria seeligeri* and *E. coli* in liquid whole egg [11]. The additional treatment of a pulsed electric field of 5.67 kV mm^−1^ and HHP of 138 MPa treatments together with the ultrasound treatment of 40 W at 55 °C in liquid whole egg were successful against *Salmonella enteritidis* [12]. Studies show the need for a treatment method which is effective against microorganisms and applies low power to minimally interact with egg components. In our prior experiment, the effect of sonication of 20–40 kHz and 180–300 W was investigated on liquid egg, albumen and yolk as well [13]. Degradation of *E. coli* was clearly detected, but inactivation solely based on ultrasonic treatment has not reached the level required for food safety [13].

The proteins in the egg determine the parameters for the heat treatment of the liquid egg products, as the aim is to avoid protein denaturation. Some proteins are also important for the technofunctional properties of the product (for example globulins, which are the only protein fraction in egg whites with good foaming properties [14]) and many egg proteins have antimicrobial properties [15]. Fotodar et al. (2003) [16] observed that E. coli (DH5alpha) can reproduce up to 49 °C and grow above 49 °C (up to 53 °C), but this is sporadic and not reproducible at this temperature range. The heat tolerance and heat sensitivity of *E. coli* bacteria depend on several factors, e.g., strain, matrix, time. By Peng et al. (2013) [17] nine *E. coli* strains were tested for heat resistance between 60 and 69 °C, with decay times varying between 3 and 132 min. Itelima et al. (2010) [18] heat-treated raw milk samples with *E. coli* O157:H7 at temperatures between 69 and 73 °C for 15 s. It was found that heat treatment at 73 °C for 15 s killed cells of this strain at all initial cell concentrations. Sörqvist (2003) [19] found that the heat treatment time required to kill 90% of the bacterial cells in the case of *E. coli* at 55 °C was 239–297 s.

The ultrasonic waves create gas bubbles in the liquid medium, which results in high temperatures and pressures when they burst out [20]. The destructive effect of ultrasound treatment on microbes is mainly due to the intracellular cavitation, meaning that micromechanical shock waves rupture the structural and functional components of cells up to the point of cell lysis [21]. The cell membrane of the microbes is thinned, local heating is observed and free radicals are produced [22]. Bacterial cells are more sensitive to heat treatment when subjected to ultrasonic treatment [23]. Increased cell death has been shown in cells subjected to combined ultrasonic and heat treatment compared to cells subjected to ultrasonic treatment alone [24]. Based on the work of Gao et al. (2016) [25], the degree of microbial inactivation during ultrasound treatments is mainly influenced by three factors: cavitation thresholds (intensity, amplitude, frequency, temperature and external pressure), the medium and the type of microorganism.

The main goal of the presented study was to determine the effect of the combination of mild heat and low-power ultrasound treatment on *E. coli* in liquid egg samples and inspection of structural changes with near-infrared (NIR) spectra analysis.

## 2. Materials and Methods

### 2.1. Materials

The pasteurized liquid egg products (albumen, yolk and whole egg) used in this study were provided by Capriovus Kft. (Szigetcsép, Hungary). The liquid egg products were made from “A” classified fresh hen eggs (determined in the 589/2008/EC regulation). Until the measurements, the liquid egg products were cold-stored at 0–4 °C in the commercially available 1 L jugs. At the start of the measurement, the contents of the required number of the jugs were poured into a bowl and gently mixed to obtain a homogeneous initial sample.

### 2.2. Ultrasonic Treatment

The effect of a combination of sonication and heat treatment on the microbiological and structural properties of liquid egg products was investigated using an ultrasonic bath (HBM Machines, MJ Mooedrecht, The Netherlands), which is capable of delivering up to 300 W of power at 20/40 kHz frequency and can heat the samples up to 55 °C.

Samples were prepared in two ways for the sonication treatment. For microbiological measurements, 180 mL of the previously homogenized samples were poured into a 200 mL glass container. For NIR measurements, 18 mL of samples were diluted with 162 mL of distilled water in order to obtain 10% (*w*/*w*) emulsions. Diluting samples is a common method in the case of evaluating NIR spectra from an aquaphotomics point of view [26,27]. In both cases, the samples were separated into four groups, based on the applied ultrasound parameters (20/40 kHz and 180/300 W equipment power) and two additional subgroups depending on the treatment time (30 and 60 min), resulting in eight ultrasound and heat-treated, an only-heat-treated and a control group (Table 1).

The ultrasound equipment was filled with 16 L of tap water to ensure sonic conductivity and this media was heated up to 55 °C. After the water bath reached the treatment temperature, the sealed 200 mL glass containers were fully submerged into the water media. The temperature of the water bath was monitored with a Pt100 temperature sensor during the whole treatment.

Preliminary experiments evaluating power absorption were carried out using distilled water. Then, 180 mL of distilled water were poured into 200 mL glass containers. One hole was made on the lid of each container for a Pt100 temperature sensor. Twelve water samples were loaded into the equipment and the temperature was monitored during the ultrasound treatment of 180 W and 300 W power at 20 kHz and 40 kHz. This measurement was carried out in four repetitions. Each time, the temperature sensor-equipped sample holder was placed at a different location inside the bath. The absorbed power was determined calorimetrically according to the following equation (Equation (1)):

(1) $$P=m\;\times \;c_{p}\;\times \;(\frac{\mathrm{dT}}{\mathrm{dt}})\;t=0$$

where m is the mass (kg), c_p_ (kJ kg^−1^ K^−1^) is the specific heat capacity of distilled water and dT/dt is the rate of temperature change during the sonication treatment, determined in 30 s intervals. The actual ultrasonic power dissipated in the liquid was calculated as 3.7 ± 0.1 and 6.9 ± 0.1 W for both frequencies, at 180 W and 300 W equipment power, respectively.

The energy dose (J) of the treatment was calculated (Equation (2)) by the multiplication of treatment duration (s) and the absorbed power (W) (Equation (2)):

D = t × P(2)

### 2.3. Preparation of Inoculation

For microbiological measurements, 180 mL of samples were inoculated with 180 µL of 1.5 × 10^8^ CFU × mL^−1^ *E. coli* (ATCC 25922) suspension. In order to determine the colony-forming unit values (CFU) of *E. coli*, a selective and differential chromogenic medium (ChromoBio COLIFORM, BioLab, Budapest, Hungary) was used. The agar indicates the presence of *E. coli* with blue color and can distinguish *E. coli* colonies from other microorganisms. 

A tenfold serial dilution was prepared after treatment and 0.1 mL of the diluted samples was transferred and spread on the surface of agar plates. The inoculated agar plates were incubated at 37 °C for 48 h. The control samples of liquid egg products inoculated with *E. coli* before sonication were investigated in the same way. Each sample was prepared in three replicates. For data analysis and visualization, the logarithm of the colony-forming unit value was considered.

### 2.4. Near-Infrared Measurements

For NIR spectral analysis, a bench=top spectrometer (MetriNIR 10-17 ST, MetriNIR Co., Budapest, Hungary) was used. The transflectance spectra were measured and collected in the wavelength range of 700–1700 nm, with the resolution of 2 nm. A water-cooled cuvette with a sample layer thickness of 0.4 mm was used to prevent data distortion due to increased temperature. The samples were prepared in triplicate and scanned in randomized order. Three consecutive scans were performed of each sample at 18 °C. 

### 2.5. Data Analysis

In order to evaluate the significant effect of ultrasound parameters and the duration of the treatment on decreasing the number of *E. coli,* two-way analysis of variance (ANOVA) was applied with Tukey-HSD test at *p* < 0.05 significance level. Homogeneity of variances was tested by Levene’s test.

In order to analyze the collected NIR spectra, the obtained data have been visualized and the outliers were removed. The Savitzky–Golay method with second-order polynomial with data frames of length 21 and without derivation was applied to smooth the spectra, followed by the Multiplicative Scatter Correction (MSC) to address the baseline shift.

Principal Component Analysis (PCA) was performed to reduce the large amount of data to a few variables, which contain the majority of the original information. In order to reduce the noise of the spectra, a wavelength range of 950–1650 nm was taken into account. PCA models were built separately for different liquid egg products.

Linear Discriminant Analysis (LDA) was performed using PCA scores in order to find a linear combination of features that may characterize the structure changes in liquid egg products during treatment. A combination of frequency and absorbed power of the ultrasound was used as class variables. Data were randomly split into three groups of equal size and threefold cross validation was performed by using two groups for calibration and one group for validation. The accuracy of prediction was calculated as the average of the confusion tables.

Analysis of microbiological data was carried out by IBM SPSS statistics 25 (IBM, Armonk, NY, USA) and Microsoft Excel 2016 (Microsoft, Redmond, Washington, USA) and NIR spectra analysis was done by RStudio 1.1.463 (RStudio Inc., Boston, MA, USA) and R 3.6.3 (R Foundation for Statistical Computing, Vienna, Austria) with the “aquap2” [28] package.

## 3. Results

### 3.1. Microbiological Measurements

**Figure 1:**
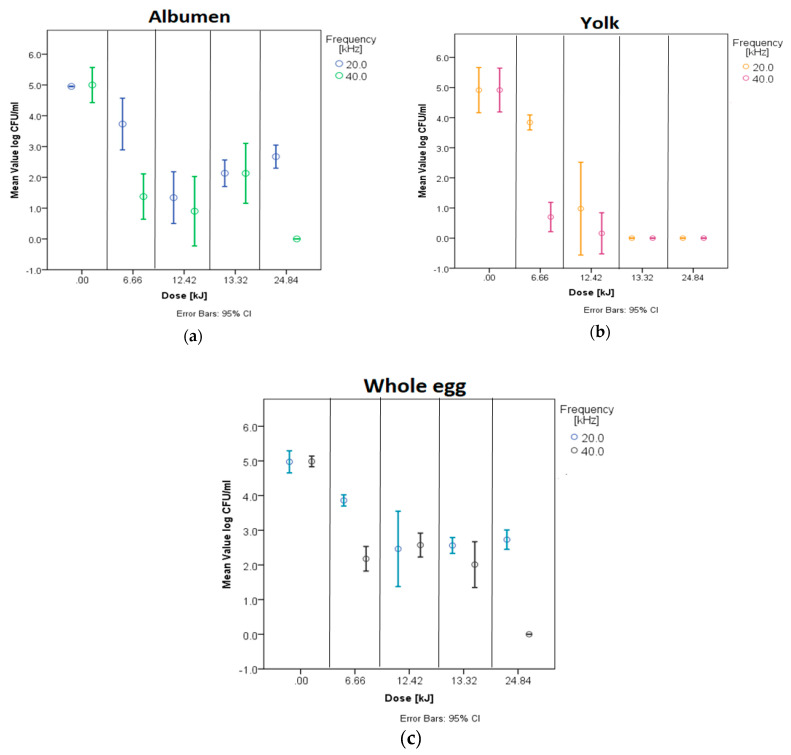
*E. coli* concentration in albumen (**a**), yolk (**b**) and whole egg (**c**) by the applied frequency and dose.

Note that 30 and 60 min long heat treatments at 55 °C alone did not cause a significant *E. coli* count reduction in all three types of egg liquid samples. The measured microbial count was close to the initial cell count (~4.9 log CFU/mL). Figure 1 shows the decrease in *E. coli* as a result of the treatment. The results showed that sonication combined with mild heat treatment was able to reduce significantly *E. coli* in the liquid egg samples below the detection limit (10 CFU × mL^−1^ ) (Figure 1). The most prominent difference was observed at 60 min treatment with 40 kHz and 6.9 W absorbed power, where the reduction was found as a 5-log CFU × mL^−1^ in whole egg liquid, yolk and albumen. In the case of yolk, the 5-log CFU × mL^−1^ reduction was obtained regardless of the applied frequency and the power of the ultrasound.

The observed data showed that every treatment was significantly (*p* < 0.05) distinguishable from the control group. Tukey test supports the assumption that the applied ultrasound power and treatment duration have a significant effect on the decrease of *E. coli* count, compared to the 55 °C heat treatment alone.

According to the Tukey test results, there was no significant difference between the effect of the levels of ultrasound frequency and power on *E. coli* count in yolk for 60 min treatments. That means that 60 min combined treatment was capable of decreasing the concentration of *E. coli* in egg yolk below 10 CFU × mL^−1^, even at the lowest applied frequency and absorbed power.

In the case of albumen, the obtained data showed that sonication frequency and the absorbed power significantly affected the reduction of *E. coli* count. There was no significant difference between 30 min and 60 min treatments.

On the other hand, a significant (*p* < 0.05) difference was observed for whole egg liquid (Table 2) concerning the duration of the treatment, but the effect of applied frequency (20 and 40 kHz) and absorbed power (3.7 and 6.9 W) levels were not significantly distinguishable from each other.

**Figure 2:**
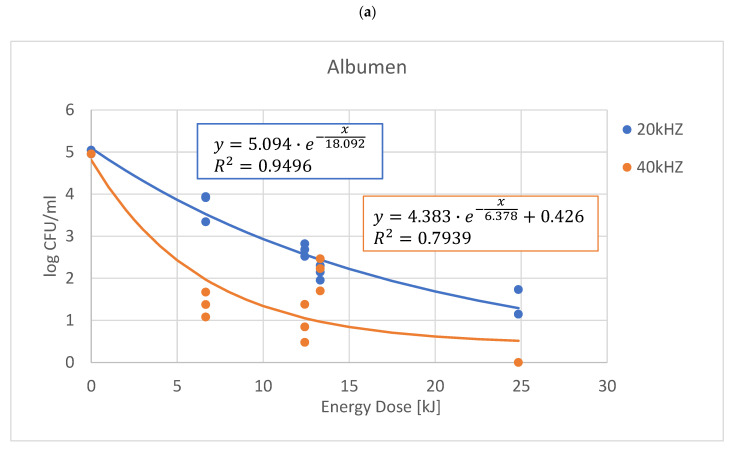

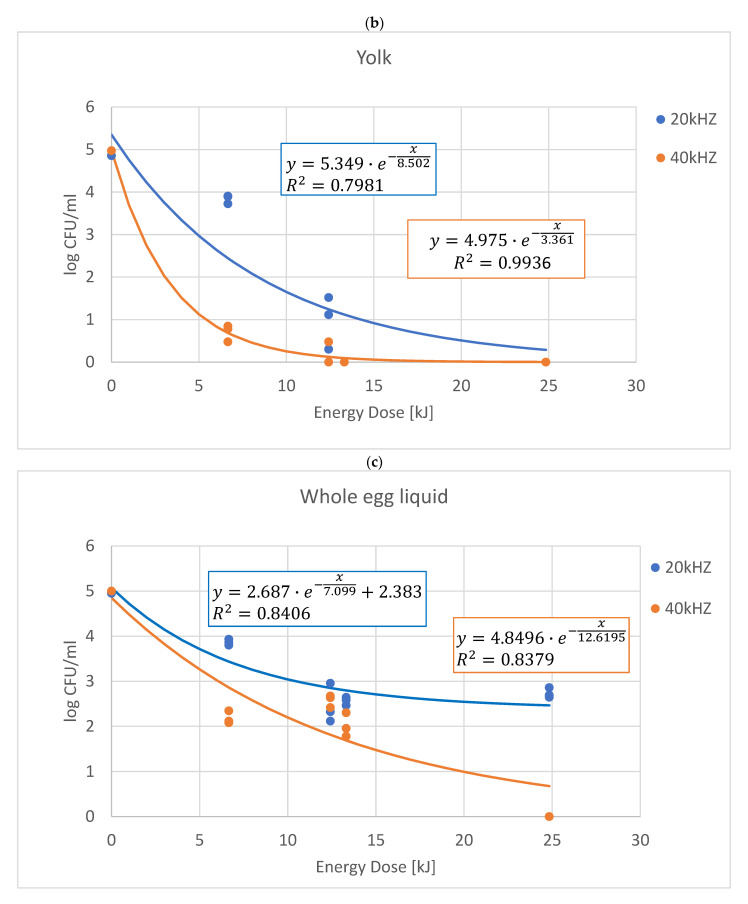
Regression of concentration (y) by the energy dose (x) of the treatment for 20 kHz and 40 kHz (**a**) albumen, (**b**) yolk, (**c**) liquid egg.

A negative exponential relationship was observed between the energy dose of ultrasound treatment and the decrease of *E. coli* count (Figure 2). The observed data showed that the highest energy dose of sonication was able to reduce the *E. coli* concentration below 10 CFU × mL^−1^ at 40 kHz treatments. However, at 20 kHz, the energy dose was not sufficient enough to reach a similar amount in the case of albumen and whole egg liquid.

Results indicated that the 20 kHz ultrasound treatment was less effective in reducing the number of *E. coli* than the 40 kHz treatment; however, there was no significant difference in the effect of ultrasound frequency in the case of yolk and whole egg liquid according to the Tukey test (*p* > 0.05).

These results are in agreement with the observation of previous experiments with milk and apple cider on the effectiveness of the combination of mild heat treatment and sonication on reducing *E. coli* concentration [5,29].

### 3.2. NIR Measurements

**Figure 3:**
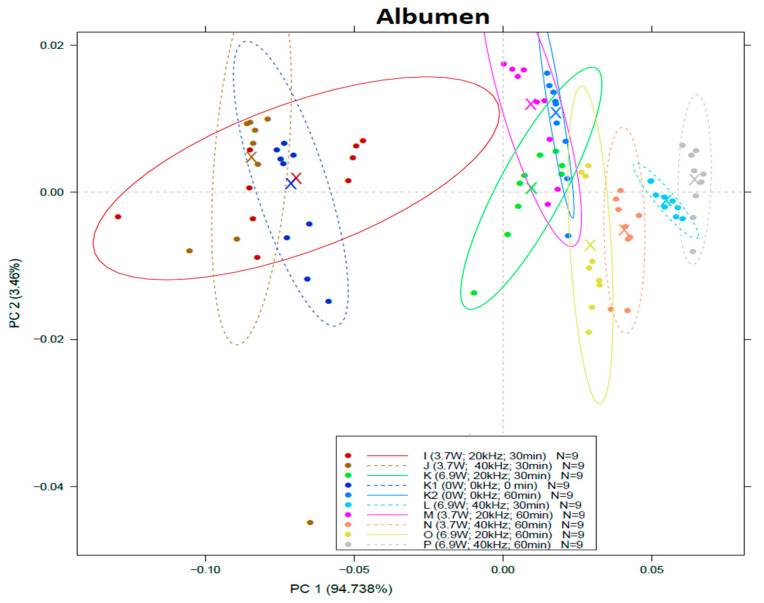
Score plot of principal component analysis (PCA) on spectral data of albumen within the range of 950–1630 nm.

PCA models (Figure 3) built from NIR spectra of liquid egg products show that the first two principal components describe a minimum 97.8% of the variance. In the case of albumen samples, the control group was not distinguishable from the samples treated at 20/40 kHz and 3.7 W for 30 min at all (group I, J). Groups treated with 20 kHz M, K, O (3.7 W and 30 min, 6.9 W and 30 min; 6.9 W and 60 min) overlapped with the heat-treated control group (K2). Groups treated at 40 kHz (L, N, P) were significantly different from both control groups.

**Figure 4:**
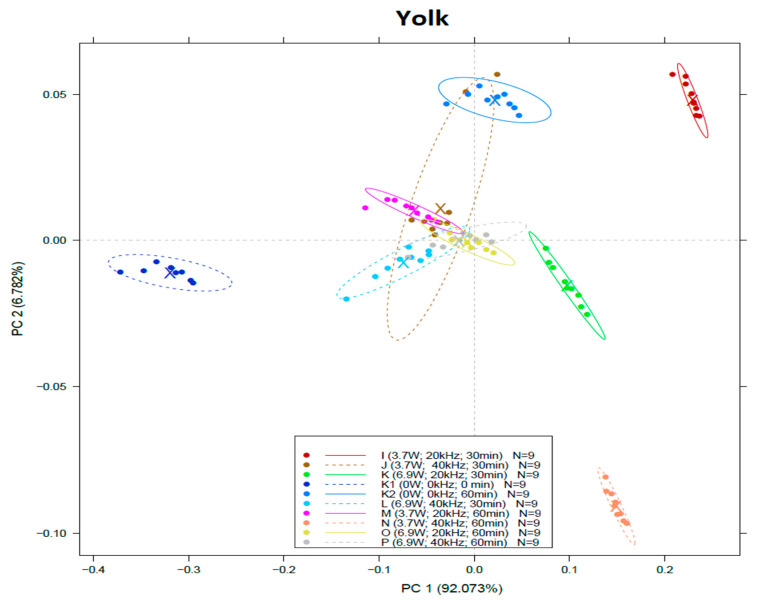
Score plot of principal component analysis (PCA) on spectral data of yolk within the range of 950–1630 nm.

The PCA plot of the NIR spectroscopy measurement of yolk can be seen Figure 4. Separation can be discovered among the control group and the treated groups, including the only-heat-treated samples along the first principal component. The samples treated at 40 kHz and 3.7 W for 30 min (Group J) show some overlapping with the only-heat-treated one (K2), but generally K2 can also be separated from the other groups along PC2. It can be seen that the spectral characteristic of Group I (3.7 kW, 20 kHz, 30 min) and N (3.7 kW, 40 kHz, 60 min) also differ significantly from others.

**Figure 5:**
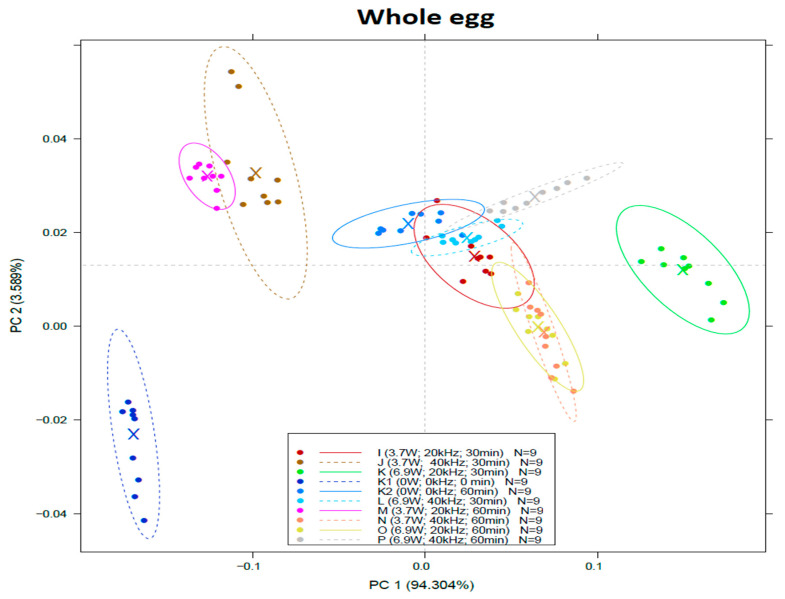
Score plot of principal component analysis (PCA) on spectral data of whole egg within the range of 950–1630 nm.

Figure 5 represents the PCA score plot of whole egg spectral characteristics. It can be seen that the control group K1 is separated from all the other groups, especially along the first principal. The samples treated at 3.7 W and 20/40 kHz for 30 and 60 min (Group M and J) are separated together from the other groups by PC1 and show similar spectral characteristics by overlapping to each other. The spectral characteristic of the heat-treated group (K2 as 0 kW, 0 kHz, 60 min) overlaps with Group I (3.7 kW, 20 kHz, 30 min) and L (6.9 kW, 40 kHz, 30 min). The separation of the groups was observed primarily along PC1, while the second principal component mostly shows the deviation within the groups.

**Figure 6:**
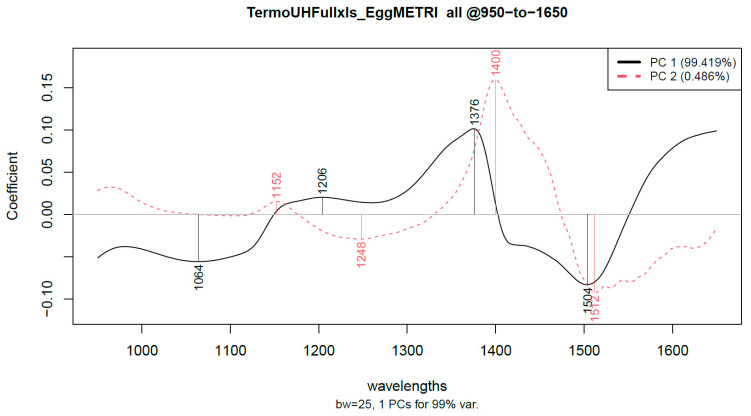
Loading plot of yolk treated at 20 kHz and 3.7 W for 60 min.

Figure 6 represents an example of the loading plots where the wavelengths highly contributing to the formation of PC1 and PC2 were acquired.

The contributing wavelengths in the formation of PC1 and PC2 for each liquid egg product are listed in Table 3. From these wavelengths, we can make further conclusions about the background of the differences in the spectral characteristics. Although the exact molecules having changed during the treatments were not determined, these wavelengths can be linked to the characteristic vibrations of chemical bonds.

Based on the observations of Tsenkova (2009), Segtnan (2001), and Bazar et al. (2015), the C-N valence vibrations are moderately strong for primary amines and are in the wavelength range of 1040–1080 nm. Approximately at 1140 and 1180 nm, the absorption bonds of secondary amines have medium intensity. The valence and deformation vibrations of the C-C bonds can be found between 1100 nm and 1300 nm. According to the observed data, the combination of ultrasonic and heat treatment is to induce changes in the C-N, C-C and the -OH characteristic vibrations [26,30,31].

There are studies, focusing on the quality changes of ultrasound and/or heat-treated egg product, reporting improved properties (foaming, emulsifying, cooking) [32,33,34]. Changes in the spectral characteristics can be related to these changes, but no such research has been published.

Linear Discriminant analysis (LDA) was performed to classify samples based on the treatment setups. The observed models showed that the treated egg groups cannot be clearly differentiated from each other. Additionally, they were mixed with the control samples in some cases.

In the case of albumen (Table 4), the classification models showed 64.72% and 63.1% accuracy for calibration and validation, respectively. The control group and the heat-treated group have a considerable overlap with the samples treated at 20/40 kHz and 3.7 W for 30 min (I and J groups). Although, group “I” has the highest overlapping with the samples treated at 40 kHz and 3.7 W for 30 min (J), indicating that the power of the sonication affected the readings. This is also supported by the fact that the samples treated by 6.9 W for 30 min (K and L) did not show similarities with these groups according to LDA.

On the other hand, the samples treated for 60 min can be distinguished clearly from the samples treated for 30 min (I, J, K, L vs. M, N, O, P). This observation indicated that the sonication power and the treatment time had a higher effect on the samples than frequency in the case of albumen (Table 4).

Linear discriminant analysis of yolk (Table 5) showed 70.53% accuracy of calibration and 71.38% on the validation model.

In this case, the control group showed no overlap with any other group. The LDA results show that, as in the case of albumen, the samples subjected to 30 min treatment differ from those in the 60 min-treated groups. Although there are some overlaps in the 60 min groups, the model showed high accuracy considering treatment time. The 30 min groups, however, could not get distinguished clearly from each other and from the heat-treated group (K2 as 0 kW, 0 kHz, 60 min). Therefore, it can be assumed that using lower ultrasound treatment duration the power and the frequency have only a slight effect on the properties of yolk. Additionally, the 55 °C heat treatment was the main contributing factor that caused changes.

On the other hand, 60 min of heat treatment (K2) had a high impact, but it seems that the different levels of frequency and the power of ultrasound had a more noticeable effect on yolk (”M”, “N”, “O”, “P” groups).

The classification accuracy of 77.23% was observed in both the calibration and validation model of whole egg samples in LDA models. The most prominent observation of this model (Table 6) is that the samples treated with ultrasound and mild heat for 60 min (M, N, O and P) are showing no overlap at all. Although, some groups treated for 30 min (I and K) show overlapping with the 60 min-treated groups. Decreasing accuracy can be observed in the case of the 30 min groups. These groups (I and J) show overlapping even with the control groups, as in the case of albumen, but the untreated group (K1) is clearly distinguishable from the other groups. According to this LDA model, the group which got only heat treatment at 55 °C shows similarity with the combined groups that were treated for 30 min (J, K and L) with ultrasound as well. These observations also support the previous assumptions that, during the 30 min combined treatment, only the heat changed most effectively the samples, but during the 60 min treatment, the power and frequency also had a noticeable effect on liquid egg quality.

Overall, LDA models showed that the duration of sonication is the most effective parameter on the spectral characteristic of the egg products. It was observed during experiments that the combined treatment was able to reduce the *E. coli* count in egg samples. However, it can alter the liquid egg properties according to NIR measurements.

## 4. Discussion

### 4.1. Microbiological Measurements

The results of the experiments showed that all treatments were significantly (*p* < 0.05) different from the control groups. The Tukey test results support the finding that the ultrasonic power and frequency used in the experiments at 55 °C has a significant effect on the reduction of *E. coli* counts compared to samples treated at 55 °C alone. Ugarte et al. (2006) also observed in their research the effect of combined ultrasound and heat treatment in reducing *E. coli* counts. They found that ultrasonic treatment at 40 °C causes cell wall deformation and shrinkage of *E. coli* cells. Treatment at 60 °C causes extensive damage and significant changes in cell morphology of *E. coli*. [29]

With the combination of ultrasound and mild heat treatment, the *E. coli* concentration was successfully reduced below the detection limit in albumen, yolk and whole egg samples.

The observed data showed that, in the case of yolk samples, the combined treatment of 60 min reduced *E. coli* counts below the detection limit regardless of the used ultrasonic power and frequency. Although, in the case of whole egg and albumen samples, this amount of reduction was only reached at 40 kHz, 6.9 W and 60 min of treatment.

In the case of all three liquid egg samples, treatment at even the lowest frequency (20 kHz) and power (180 W) for 30 min significantly reduced the initial *E. coli* count.

A negative exponential relationship was observed between the energy dose of ultrasound treatment and the decrease of *E. coli* count. Results indicated that the 20 kHz ultrasound treatment was less effective in the *E. coli* number reduction than the 40 kHz treatment. However, there was no significant difference in the effect of ultrasound frequency in the case of yolk and whole egg liquid according to the Tukey test (*p* > 0.05).

### 4.2. NIR Measurements

Using NIR spectral analysis, we were able to detect the changes caused by the combined ultrasound and mild heat treatment in liquid samples.

The PCA analysis of the observed NIR spectra showed the effect of the treatment properties on spectral characteristics of the liquid egg products. According to these observations, the control group (K1) differs from the treated samples in the case of yolk and whole egg.

The wavelengths contributing to the first two principal components of the PCA analysis were obtained and examined. These wavelengths are related to molecular bonds that contribute to the changes resulting from the treatment. These wavelengths are also related to different bonds in the egg liquid. Therefore, it is assumed that these wavelength bonds were modified during the combined treatment.

Due to the combined ultrasound and thermal treatment, the contributing wavelengths show that C-C, C-N, -OH, N-H bonds were altered in both albumen, yolk and whole egg liquid samples.

Based on the LDA results, the most prominent observation was in the case of whole egg samples, where the 60 min treatment groups (M, N, O, P) showed no overlapping with the other groups.

## 5. Conclusions

According to our results, it can be stated that the combined application of ultrasound and mild heat treatment (40 kHz, 300 W, 60 min and 55 °C) decreased the *E. coli* number of the liquid egg samples below the detection limit.

Although, the exact molecules were not determined, a connection was found between NIR wavelengths and molecular bond vibrations. This means that NIR spectroscopy can be a useful tool to determine alteration in egg samples due to the combination of heat and ultrasound treatments, which supports near-infrared spectroscopy as a non-destructive food monitoring method.

## Figures and Tables

**Table 1:** Egg sample groups depending on the applied treatment.

	Frequency (kHz)	Equipment Power (W)	Duration (min)	Temperature (°C)
I	20	180	30	55
J	40	180	30	55
K	20	300	30	55
L	40	300	30	55
M	20	180	60	55
N	40	180	60	55
O	20	300	60	55
P	40	300	60	55
K2	0	0	60	55
K1	0	0	0	0

**Table 2:** Two-way ANOVA results of whole egg samples considering treatment time and frequency.

Tests of Between-Subjects Effects ^a^					
Dependent Variable: Value log CFU/mL					
Source	Type III Sum of Squares	df	Mean Square	F	Sig.
Corrected Model	84.484 ^b^	5	16.897	27.048	0.000
Intercept	96.291	1	96.291	154.141	0.000
Time	72.719	2	36.359	58.203	0.000
Frequency	2.082	1	2.082	3.333	0.082
Time × Frequency	6.925	2	3.462	5.542	0.011
Error	13.743	22	0.625		
Total	146.305	28			
Corrected Total	98.228	27			

^a^ Group = Whole Egg. ^b^ R Squared = 0.753, (Adjusted R Squared = 0.722).

**Table 3:** Contributing wavelengths of the PCA models.

Egg Product	Treatment Setup	Wavelengths			
		C-N	C-C	-OH	N-H
Albumen	55 °C only	1068	1152, 1248	1386, 1460	-
	20 kHz, 3.7 W	1068, 1086	1156, 1266	1366, 1466, 1530	-
	20 kHz, 6.9 W	1038	1146, 1288	1324, 1370, 1434, 1550	-
	40 kHz, 3.7 W	1082	1148, 1166, 1266	1344, 1388, 1462, 1550	-
	40 kHz, 6.9 W	1052	1158	1338, 1454	1562
Yolk	55 °C only	1066	1206	1368, 1412, 1512	-
	20 kHz, 3.7 W	1064	1152, 1206, 1248	1376, 1400, 1504, 1512	-
	20 kHz, 6.9 W	1046, 1068	1156, 1206	1372, 1384, 1410, 1514	-
	40 kHz, 3.7 W	1066	1154, 1206, 1224	1370, 1396, 1416, 1510	-
	40 kHz, 6.9 W	1064	1206	1374, 1398, 1416, 1506	1624
Whole egg	55 °C only	1060	1196	1380, 1484	-
	20 kHz, 3.7 W	1062	1188, 1206	1308, 1380, 1494, 1556	-
	20 kHz, 6.9 W	976, 1060	1112, 1190, 1210	1382, 1496	-
	40 kHz, 3.7 W	1060	1154, 1188	1382, 1498, 1544	1626
	40 kHz, 6.9 W	1056, 1060	1192	1376, 1398, 1494	1554, 1608, 1624

**Table 4:** Classification accuracy of linear discriminant analysis of albumen samples, %.

Albumen	K1 Control	K2 (0 W; 0 kHz; 60 min)	I (3.7 W; 20 kHz; 30 min)	J (3.7 W; 40 kHz; 30 min)	K (6.9 W; 20 kHz; 30 min)	L (6.9 W; 40 kHz; 30 min)	M (3.7 W; 20 kHz; 60 min)	N (3.7 W; 40 kHz; 60 min)	O (6.9 W; 20 kHz; 60 min)	P (6.9 W; 40 kHz; 60 min)
K1	**49.44**	2.31	3.01	2.75	0	0	0	0	0	0
K2	2.42	**50**	5.58	16.67	0	0	0	0	0	0
I	44.62	42.07	**50**	11.08	0	0	0	0	0	0
J	3.52	5.62	41.41	**63.92**	0	0	0	0	0	0
K	0	0	0	0	**88.92**	45.26	2.75	0	0	8.33
L	0	0	0	0	0	**50**	0	38.92	0	0
M	0	0	0	0	0	4.74	**97.25**	0	0	0
N	0	0	0	0	8.33	0	0	**50**	0	0
O	0	0	0	0	0	0	0	0	**100**	41.67
P	0	0	0	5.58	2.75	0	0	11.08	0	**50**

**Table 5:** Classification accuracy of linear discriminant analysis of yolk samples, %.

Yolk	K1 Control	K2 (0 W; 0 kHz; 60 min)	I (3.7 W; 20 kHz; 30 min)	J (3.7 W; 40 kHz; 30 min)	K (6.9 W; 20 kHz; 30 min)	L (6.9 W; 40 kHz; 30 min)	M (3.7 W; 20 kHz; 60 min)	N (3.7 W; 40 kHz; 60 min)	O (6.9 W; 20 kHz; 60 min)	P (6.9 W; 40 kHz; 60 min)
K1	**100**	0	0	0	0	0	0	0	0	0
K2	0	**72.25**	33.33	44.46	0	38.92	0	0	0	5.58
I	0	27.75	**50**	0	16.67	0	0	0	0	0
J	0	0	0	**52.79**	0	11.08	0	0	0	5.58
K	0	0	16.67	0	**50**	0	0	0	0	0
L	0	0	0	0	33.33	**50**	0	0	0	0
M	0	0	0	2.75	0	0	**100**	50	0	0
N	0	0	0	0	0	0	0	**50**	0	0
O	0	0	0	0	0	0	0	0	**100**	0
P	0	0	0	0	0	0	0	0	0	**88.84**

**Table 6:** Classification accuracy of linear discriminant analysis of whole egg samples, %.

Whole Egg	K1 Control	K2 (0 W; 0 kHz; 60 min)	I (3.7 W; 20 kHz; 30 min)	J (3.7 W; 40 kHz; 30 min)	K (6.9 W; 20 kHz; 30 min)	L (6.9 W; 40 kHz; 30 min)	M (3.7 W; 20 kHz; 60 min)	N (3.7 W; 40 kHz; 60 min)	O (6.9 W; 20 kHz; 60 min)	P (6.9 W; 40 kHz; 60 min)
K1	**91.67**	0	27.75	44.42	0	0	0	0	0	0
K2	0	**38.92**	0	0	8.33	22.25	0	0	0	0
I	2.75	0	**47.25**	0	0	0	0	0	0	0
J	5.58	47.25	0	**55.58**	0	0	0	0	0	0
K	0	11.08	0	0	**61.08**	0	0	0	0	0
L	0	2.75	19.42	0	13.92	**77.75**	0	0	0	0
M	0	0	0	0	0	0	**100**	0	0	0
N	0	0	5.58	0	0	0	0	**100**	0	0
O	0	0	0	0	16.67	0	0	0	**100**	0
P	0	0	0	0	0	0	0	0	0	**100**

## References

[1] Bhargava N., Mor R.S., Kumar K., Sharanagat V.S. (2021). Advances in application of ultrasound in food processing: A review. Ultrason. Sonochem..

[2] Veeraapandian S., Sawant S.N., Doble M. (2012). Antibacterial and antioxidant activity of protein capped silver and gold nanoparticles synthesized with Escherichia coli. J. Biomed. Nanotechnol..

[3] Lee H., Zhou B., Liang W., Feng H., Martin S.E. (2009). Inactivation of Escherichia coli cells with sonication, manosonication, thermosonication, and manothermosonication: Microbial responses and kinetics modeling. J. Food Eng..

[4] Kang D., Jiang Y., Xing L., Zhou G., Zhang W. (2017). Inactivation of Escherichia coli O157:H7 and Bacillus cereus by power ultrasound during the curing processing in brining liquid and beef. Food Res. Int..

[5] Görgüç A., Gençdağ E., Okuroğlu F., Yılmaz F.M., Bıyık H.H., Öztürk Köse S., Ersus S. (2021). Single and combined decontamination effects of power-ultrasound, peroxyacetic acid and sodium chloride sanitizing treatments on Escherichia coli, Bacillus cereus and Penicillium expansum inoculated dried figs. LWT.

[6] D’AMICO D.J., SILK T.M., WU J., GUO M. (2006). Inactivation of Microorganisms in Milk and Apple Cider Treated with Ultrasound. J. Food Prot..

[7] Lee H., Kim H., Cadwallader K.R., Feng H., Martin S.E. (2013). Sonication in combination with heat and low pressure as an alternative pasteurization treatment—Effect on Escherichia coli K12 inactivation and quality of apple cider. Ultrason. Sonochem..

[8] Wu X., Narsimhan G. (2017). Synergistic effect of low power ultrasonication on antimicrobial activity of melittin against Listeria monocytogenes. LWT.

[9] Nguyen Huu C., Rai R., Yang X., Tikekar R.V., Nitin N. (2021). Synergistic inactivation of bacteria based on a combination of low frequency, low-intensity ultrasound and a food grade antioxidant. Ultrason. Sonochem..

[10] Bi X., Wang X., Chen Y., Chen L., Xing Y., Che Z. (2020). Effects of combination treatments of lysozyme and high power ultrasound on the Salmonella typhimurium inactivation and quality of liquid whole egg. Ultrason. Sonochem..

[11] Lee D.-U. (2009). Effects of Combination Treatments of Nisin and High-intensity Ultrasound with High Pressure on the Functional Properties of Liquid Whole Egg. Food Sci. Biotechnol..

[12] Huang E., Mittal G.S., Griffiths M.W. (2006). Inactivation of Salmonella enteritidis in liquid whole egg using combination treatments of pulsed electric field, high pressure and ultrasound. Biosyst. Eng..

[13] Nagy D., Felfoldi J., Taczmanne Bruckner A., Mohacsi-Farkas C., Bodor Z., Kertesz I., Nemeth C., Zsom-Muha V. (2021). Determining Sonication Effect on E. coli in Liquid Egg, Egg Yolk and Albumen and Inspecting Structural Property Changes by Near-Infrared Spectra. Sensors.

[14] Csurka T., Szücs F., Csehi B., Friedrich L.F., Pásztor-Huszár K. (2021). Analysis of several techno-functional and sensory attributes upon egg allergen ingredient substitution by blood plasma powder in sponge cake. Prog. Agric. Eng. Sci..

[15] Németh C. (2012). Low Temperature Heat Treatment of Liquid Eggs.

[16] Fotadar U., Zaveloff P., Terracio L. (2005). Growth of Escherichia coli at elevated temperatures. J. Basic Microbiol..

[17] Peng S., Hummerjohann J., Stephan R., Hammer P. (2013). Short communication: Heat resistance of Escherichia coli strains in raw milk at different subpasteurization conditions. J. Dairy Sci..

[18] Itelima J.U., Agina S.E. (2011). Effect of heat treatment on the survival of Escherichia Coli O157: H7 in raw milk. Glob. J. Pure Appl. Sci..

[19] Sörqvist S. (2003). Heat Resistance in Liquids of *Enterococcus* spp., *Listeria* spp., *Escherichia coli*, *Yersinia enterocolitica*, *Salmonella* spp. and *Campylobacter* spp.. Acta Vet. Scand..

[20] Vollmer A.C., Kwakye S., Halpern M., Everbach E.C. (1998). Bacterial Stress Responses to 1-Megahertz Pulsed Ultrasound in the Presence of Microbubbles. Appl. Environ. Microbiol..

[21] Butz P., Tauscher B. (2002). Emerging technologies: Chemical aspects. Food Res. Int..

[22] Piyasena P., Mohareb E., McKellar R.C. (2003). Inactivation of microbes using ultrasound: A review. Int. J. Food Microbiol..

[23] Ordoñez J.A., Sanz B., Hernandez P.E., Lopez-Lorenzo P. (1984). A note on the effect of combined ultrasonic and heat treatments on the survival of thermoduric streptococci. J. Appl. Bacteriol..

[24] Earnshaw R.G., Appleyard J., Hurst R.M. (1995). Understanding physical inactivation processes: Combined preservation opportunities using heat, ultrasound and pressure. Int. J. Food Microbiol..

[25] Gao S., Lewis G., Hemar Y. (2016). Ultrasonic Inactivation of Microorganisms. Handbook of Ultrasonics and Sonochemistry.

[26] Bazar G., Kovacs Z., Tanaka M., Furukawa A., Nagai A., Osawa M., Itakura Y., Sugiyama H., Tsenkova R. (2015). Water revealed as molecular mirror when measuring low concentrations of sugar with near infrared light. Anal. Chim. Acta.

[27] Kovacs Z., Pollner B., Bazar G., Muncan J., Tsenkova R. (2020). A Novel Tool for Visualization of Water Molecular Structure and Its Changes, Expressed on the Scale of Temperature Influence. Molecules.

[28] Kovacs Z., Pollner B. Aquaphotomics-Software R-Package “aquap2”. Proceedings of the Understanding Water in Biology 2nd International Symposium.

[29] Ugarte-Romero E., Feng H., Martin S.E., Cadwallader K.R., Robinson S.J. (2006). Inactivation of Escherichia coli with Power Ultrasound in Apple Cider. J. Food Sci..

[30] Tsenkova R. (2009). Introduction Aquaphotomics: Dynamic spectroscopy of aqueous and biological systems describes peculiarities of water. J. Near Infrared Spectrosc..

[31] Segtnan V.H., Šašić Š., Isaksson T., Ozaki Y. (2001). Studies on the Structure of Water Using Two-Dimensional Near-Infrared Correlation Spectroscopy and Principal Component Analysis. Anal. Chem..

[32] Nagy D., Zsom-Muha V., Németh C., Felföldi J. (2021). Sonication effect on foam properties of egg white. Prog. Agric. Eng. Sci..

[33] Jovanovic J.R., Stefanovic A.B., Sekuljica N.Z., Jakovetic Tanaskovic S.M., Dojcinovic M.B., Bugarski B.M., Knezevic-Jugovic Z.D. (2016). Ultrasound Pretreatment as an Useful Tool to Enhance Egg White Protein Hydrolysis: Kinetics, Reaction Model, and Thermodinamics. J. Food Sci..

[34] Stefanovic A.B., Jovanovic J.R., Dojcinovic M.B., Levic S.M., Nedovic V.A., Bugarski B.M., Knezevic-Jugovic Z.D. (2017). Effect of the Controlled High-Intensity Ultrasound on Improving Functionality and Structural Changes of Egg White Proteins. Food Bioprocess Technol..

